# Behavioural, Mucosal and Systemic Immune Parameters in HIV-infected and Uninfected Injection Drug Users

**DOI:** 10.4172/2155-6105.1000257

**Published:** 2015

**Authors:** Saurabh Mehandru, Sherry Deren, Sung-Yeon Kang, Angela Banfield, Aakash Garg, Donald Garmon, Melissa LaMar, Teresa H Evering, Martin Markowitz

**Affiliations:** 1 Division of Gastroenterology, Department of Medicine, Mount Sinai School of Medicine, New York, USA; 2 Center for Drug Use and HIV Research, New York University College of Nursing, New York, USA; 3 Aaron Diamond AIDS Research Center and The Rockefeller University, New York, USA

**Keywords:** Injection drug use, Immune activation, Mucosal, HIV

## Abstract

**Objective:**

Injection drug use (IDU) remains a major risk factor for HIV-1 acquisition. The complex interplay between drug use, non-sterile injection, and Hepatitis C remains poorly understood. We conducted a pilot study to determine the effect of IDU on immune parameters among HIV-uninfected and -infected individuals. We hypothesized that IDU could further augment immunological changes associated with HIV-1 infection, which could in turn affect HIV pathogenesis

**Methods:**

HIV-uninfected and -infected subjects with IDU, and non-IDU controls were recruited to obtain socio-demographic and drug-related behaviours. Blood (PBMC) and mucosal (MMC) mononuclear cells were analysed for cellular markers of immune activation (CD38 and Ki67). Serum ELISA was performed to determine levels of soluble CD14, a marker of immune activation.

**Results:**

No significant quantitative differences in CD4^+^ and CD8^+^ T cell levels were observed between IDU and non-IDU subjects when accounting for the presence of HIV-1 infection. However, increased levels of cellular and soluble markers of immune activation were documented in cells and plasma of HIV-uninfected IDU subjects compared to non-injectors. Additionally, sharing of injection paraphernalia was related to immune activation among HIV-uninfected IDU subjects.

**Conclusion:**

IDU, with or without HIV-1 infection, results in a significant increase in immune activation in both the peripheral blood and the GI tract. This may have significant impact on HIV transmission, pathogenesis, and immunologic responses to combination antiviral therapy. This study provides compelling preliminary results which in turn support larger studies to better define the relationship between IDU, infection with HIV-1, co-infection with Hepatitis C and immunity.

## Background

The devastating consequences of untreated HIV-1 infection on the human immune system have spurred investigations aimed at better understanding disease pathogenesis. Studies of acute and early CCR5-tropic HIV-1 infection in man and SIV_mac_ infection in rhesus macaques have demonstrated that CD4^+^ T cells at mucosal sites, particularly within the gastrointestinal (GI) tract, are preferentially targeted and depleted [1–4]. In rhesus macaques rapid progression to simian AIDS has been associated with failure to regenerate critical effector immune cells at the tissue/environment interface, whereas in the remainder of infected animals, a striking proliferative response presumably derived from the naïve and central memory T cell compartment is observed [5]. A cross-sectional study suggested that this early and non-resolving GI tract lesion allows for the translocation of microbial products such as lipopolysaccharide (LPS) into the systemic circulation, contributing to immune activation and enhancing clinical progression [6]. More recently it has been suggested that there is selective depletion of the Th17 cells from the GI tract- and that the loss of these cells may result in loss of mucosal integrity, translocation of microbial products, immune activation and subsequent CD4^+^ T cell loss [7].

These findings have emerged primarily from studying non-injection drug using HIV-1 infected cohorts, predominantly men who have sex with men (MSM). Relatively few studies have included injection drug users (IDU), though it is estimated that among those currently living with HIV/AIDS, IDU was the third leading category of HIV transmission after male-to-male and heterosexual contact [8]. Biomedical studies of subjects with IDU are underrepresented in the literature. A variety of factors, which include concerns about recruiting and retaining IDUs in clinical trials are responsible. In order to expand the findings derived from studies of MSM to others at risk and infected populations, it is essential to examine these relationships for injection drug users (IDUs) of both sexes. It also remains unknown whether active injection drug use when superimposed on HIV-1 infection accelerates the loss of CD4^+^ T cell in the GI tract and periphery due to increased levels of activation due to non-specific antigenic stimulation.

Although behavioural factors are believed central to IDU-mediated HIV transmission, the relationship between injecting behaviours and disease pathogenesis are less clear. There are disparities in correlating behaviours with outcomes. For example, Mientjes et al. found a lower rate of HIV-1 related disease progression among those who frequently borrowed injection equipment [9]. Whereas, Donahoe et al. [10] and Krol et al. [11] found no relationship between the rate of CD4^+^ cell decline, and frequency of injection drug use respectively. Thus, further research on behavioural and immunological correlates of IDU on HIV pathogenesis is clearly indicated.

We performed an exploratory study using an integrated biomedical/behavioural multidisciplinary approach to examine the effect of injection drug use on gut associated lymphoid tissue (GALT) in the setting of HIV-1 infection. The specific aims of this study were to 1) to determine the effect of injection drug use on the degree of CD4^+^ T cell depletion in the GI tract and whether this effect is modified by HIV, 2) explore the effect of IDU on systemic immune activation and investigate its impact on the mucosal immune system in the absence and presence of chronic untreated HIV-1 infection and 3) ascertain the relationship between IDU-related behavioural factors and measures of HIV disease, including the degrees of CD4^+^ T cell depletion and the levels of HIV-1 RNA. We believe this to be the first description of such in HIV-1 infected and uninfected IDUs.

## Methods

### Study participants

This pilot study was conducted over a two-year period from 2009 to 2011. Four groups of participants were recruited: (a) Group 1: healthy volunteers (non-IDUs, HIV-uninfected, n=13); (b) Group 2: HIV-1 uninfected IDUs, n=19; (c) Group 3: HIV-1 infected, viremic, non-IDUs, n=8 and (d) Group 4: HIV-1 infected, viremic, IDUs n=10. Recruitment occurred at needle exchange programs (NEPs), other harm reduction programs, non-government organizations (NGOs) serving HIV-infected persons, Infectious Disease clinics, and NYC-based research projects conducting studies with IDUs or HIV-infected persons. Recruitment for the healthy volunteers occurred through the Rockefeller University Hospital Office of Research Support. Criteria for participation of IDUs included: current injection drug use (atleast 3 times/week), age 18–65. Additional criteria for HIV- infected participants included the presence of viremia and for non-IDU controls, no lifetime injection and no recent (past 5 years) non-injection use of heroin or cocaine. We initially recruited the IDU groups and subsequently identified the non-IDU controls and matched when feasible for gender, race/ethnicity, and age (+/− 5 years).

### Enrolment procedures

At the first contact the project was briefly described to potential participants, including incentives and information about the sigmoidoscopy procedure. Experienced recruiters verified current injection drug use through examination of track marks and questions regarding injection practices. Those meeting the initial criteria were scheduled for two visits. At the first visit, the informed consent was orally administered with use of an audiotape, and included a detailed one-on-one discussion to ensure that the participant understood the protocol procedures and thus made an informed decision to participate. After written consent was obtained, a brief computer-assisted interview was administered, that collected data on (a) socio-demographic characteristics, including age, gender, sexual orientation, race/ethnicity, residential status; (b) injection drug use behaviours: age first injected and current injection behaviour, including types of drugs, frequency, syringe sharing, injection equipment sharing (cookers, cottons, rinse water); and (c) non-injection drug use (alcohol and other substances) including history of use and current frequency and routes of administration.

After the interview was completed, medical history and physical exam were conducted to determine eligibility for study. The sigmoidoscopy was scheduled at this time, and generally followed approximately 2 weeks from screening. After results of phlebotomy and urine collection were reviewed, inclusion and exclusion criteria were assessed (e.g., detectable plasma viral load for the HIV-infected participants). On the morning of the procedure 2 fleet enemas were administered and a flexible sigmoidoscopy with biopsy was performed. All protocols were reviewed and approved by the Rockefeller University and New York University Institutional Review Boards.

### Peripheral blood and gastrointestinal sample acquisition

Peripheral blood and recto-sigmoid colonic mucosal tissue were collected from all participants and processed as previously described [3].

### Flow cytometry

Cell surface expression of lymphocyte antigens was performed by monoclonal antibody staining of freshly isolated MMCs and PBMCs, followed by flow cytometry using a BDLSRII instrument (Becton-Dickinson, Palo Alto, California, United States) with analysis using FlowJo software (TreeStar). Monoclonal antibodies used in this study included: anti-human CD3-Pacific Blue (PB) (clone UCHT1) (BD Pharmingen), anti-human CD4-Alexa700 (clone RPA T4) ((BD Pharmingen), anti-human CD8 APC-Cy7 (clone SK1) (BD Pharmingen), anti-human CD38-PE (clone HIT2) (BD Pharmingen), and anti-human Ki67-PE (clone B56) (BD Pharmingen), and the appropriate isotype controls. Flow cytometric analyses were performed as previously described [3].

### sCD14 quantification

Human sCD14 Quantikine ELISA kit was used to determine the levels of sCD14 in the plasma of group 1–4 subjects using manufacturer’s protocol.

### Statistical methodology

Values are expressed as mean ± standard deviation. Statistical comparisons were made between PBMCs and MMCs from individuals using a paired t-test. Statistical comparisons were made between HIV uninfected and HIV infected IDU and control participants using a two-sample, unequal variance t-test. For analysis of the behavioural data, statistical differences between groups were tested using Fisher’s exact tests for categorical variables and one-way ANOVA and two-tailed t-tests for continuous variables. Relationships between behavioural variables and immune activation were examined using Pearson correlation coefficients. All reported p-values were two-sided at the 0.05 significance level using SPSS 21.0 for Windows software (SPSS, Chicago, Illinois, United States).

## Results

### Patient characteristics

A total of 401 potential participants contacted the recruiters. Based on telephone screening, approximately half were not eligible because they were not a current IDU or, after the targeted number of uninfected IDUs was reached, they were not HIV-1 infected (49%). An additional 18% did not return calls or were not interested upon hearing more about the study. A total of 79 (20%) were eligible for formal screening at Rockefeller University Hospital. Twenty-two failed to qualify for the study. Reasons included hepatic decompensation, lack of venous access, and HIV-1 RNA levels below detection. Of those eligible for the sigmoidoscopy (57), 88% successfully completed the procedure. No procedure- related grade 2 or greater adverse events were reported, and participant response was generally favourable.

A total of 29 IDUs (19 HIV-uninfected, 10 HIV-infected) and 21 non-IDUs (13 uninfected and 8 infected) completed the study (Table 1). Participants were primarily male with average age in the late 30s and early 40s, and primarily Hispanic or African American. While there was no significant difference across groups in gender or mean age, the HIV-infected non-IDUs were more likely to be Black (and less likely Hispanic), and the IDU groups were less likely to have completed High School. The mean HIV viral load in HIV infected non-IDUs was 58,409 copies/ml plasma (range- 13,571–214696 copies/ml) and the mean HIV viral load in the HIV infected IDUs was 63,046 copies/ml plasma (range- 7,259–111,609 copies/ml) (p=0.8). All participants were screened for antibodies to hepatitis C virus (HCV). None of 13 patients in group 1, 14 of 19 patients in group 2, 1 of 9 patients in group 3 and 9 of 10 patients in group 4 tested positive serologically for HCV (Table 1). Drug use and injection-related behavioural variables on the two IDU groups were elicited during the screening visit (Table 2). Both groups had substantial duration of IDU (means of 13.5 years for the HIV-uninfected and 19.6 for the HIV-infected group), and reported high frequencies of injection during the prior 30 days. Heroin injection was reported by the majority of IDUs and HIV-infected IDUs were more likely to inject speedball (combination of heroin and cocaine) and less likely to inject other substances (p<0.05). Those who were HIV-infected reported lower rates of recent sharing of syringes (20% vs. 58%; p<0.05), and both groups were similar in sharing of other injection-related paraphernalia. The HIV-infected IDUs reported higher rates of non-injection heroin use and the majority among both groups reported use of methadone.

### Levels of T cell subsets in the peripheral blood and the gastrointestinal tract

We quantified T cell subsets in the peripheral blood mononuclear cells (PBMC) and mucosal mononuclear (MMC) of study volunteers using paired samples derived from the peripheral blood and the gastrointestinal (GI) tract respectively where live (Aqua^−^) CD3^+^ T cells are assessed for CD4^+^ and CD8^+^ lymphocyte subsets. The frequency of CD4^+^ PBMC and MMC was comparable in HIV-uninfected, group 1 volunteers (57.6% ± 6.4 CD4^+^ PBMC and 65.1% ± 4.8 CD4^+^ MMC) and HIV- uninfected group 2 volunteers (55.2% ± 11.5 CD4^+^ PBMC, p=0.5 and 65.0% ± 10.1 CD4^+^ MMC, p=0.9 respectively). In contrast, as expected, compared to group 1 healthy subjects, CD4^+^ T cells were significantly depleted from the peripheral blood and GI tract of HIV-infected, non-IDU, group 3 (14.6% ± 8.4 CD4^+^ PBMC, p<0.001 and 12.3% ± 9.3 CD4^+^ MMC, p<0.001) as well as HIV-infected IDU, group 4 subjects (25.6% ± 12.1 CD4^+^ PBMC, p<0.001 and 12.3% ± 9.3 CD4^+^ MMC, p<0.001). No significant differences were observed in the percentages of CD4^+^ MMC (p=0.5) between the 2 HIV-1 infected groups although a non-significant trend was seen with higher percentage of CD4+ PBMC in HIV-1 infected IDU compared to HIV-1 infected non-IDU controls (p=0.05).

**Figure 1:**
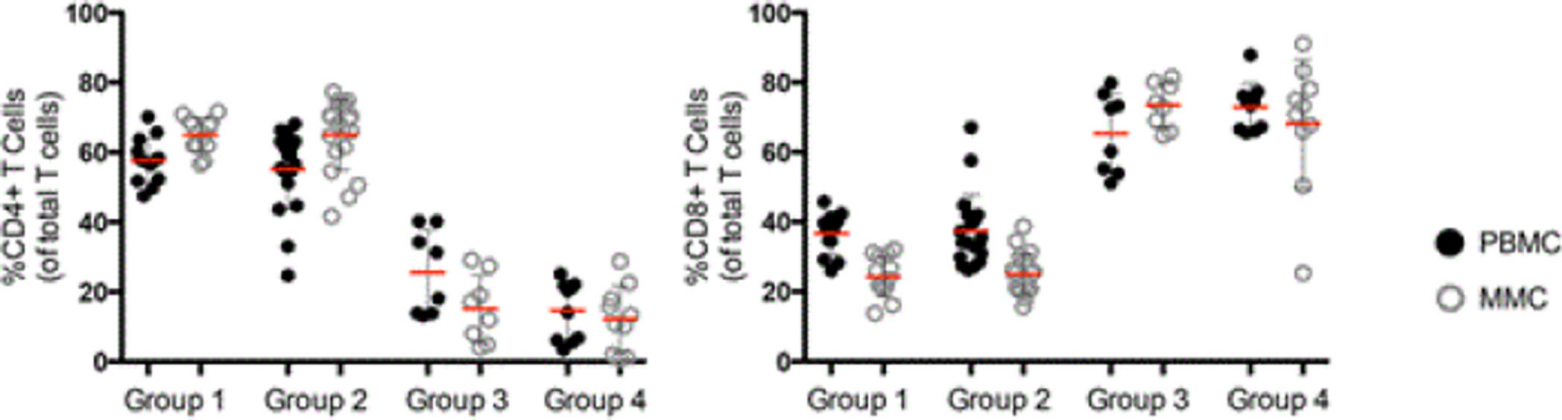
Impact of IDU on T cell subsets in the peripheral blood and the GI tract. (a) Percentage of CD4^+^ T cells (left panel) and CD8^+^ T cells (right panel) from Group 1 (non-IDUs, HIV-uninfected, n=13); Group 2 (HIV-1 uninfected IDUs, n=19); Group 3 (HIV-1 infected, viremic, non-IDUs, n=8) and Group 4 (HIV-1 infected, viremic, IDUs n=10). PBMCs are depicted by dark circles and MMC by open grey circles. Red horizontal bars represent the mean per group examined.

Analogously, CD8^+^ T cells were proportionally significantly increased in HIV-infected, non-IDU group 3 subjects (65.3% ± 11.4 CD8^+^ PBMC, p<0.001 and 73.4% ± 6.4 CD8^+^ MMC, p<0.001) and HIV-infected IDU group 4 subjects (72.9% ± 6.9 CD8^+^ PBMC, p<0.001 and 68.1% ± 18.5 CD8^+^ MMC, p<0.001) compared to healthy uninfected group 1 subjects (36.7% ± 5.9 CD8^+^ PBMC and 24.3% ± 5.8 CD8^+^ MMC). No significant differences were observed in the CD8^+^ T cell percentages between healthy volunteers and HIV-uninfected IDUs (37.4% ± 10.4 CD8^+^ PBMC, p=0.8 and 25.1% ± 5.9 CD8^+^ MMC, p=0.7). Similarly, no significant differences were observed in the percentage of CD8^+^ T cells in the blood or GI tract between group of HIV-1 infected subjects (p=0.1 and p=0.4 respectively) (Figure 1).

### Cellular markers of immune activation and proliferation in the peripheral blood and gastrointestinal tract

Having observed no significant numerical differences in T cells between IDU and non-IDU subjects, we next studied qualitative parameters, comparing markers of immunological activation (CD38) and proliferation (Ki67) in these groups.

**Figure 2:**
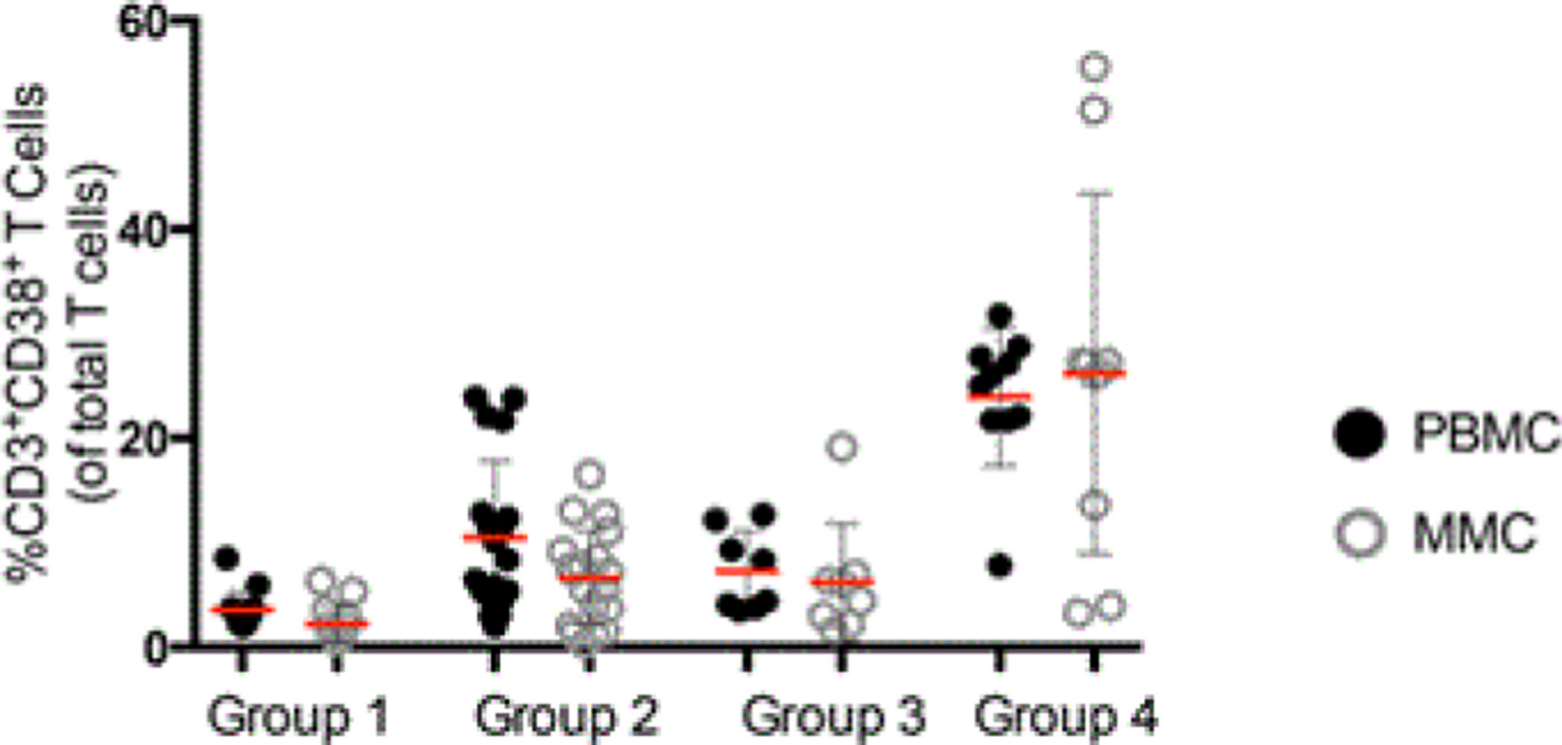
Cumulative data comparing the percentage of CD3^+^CD38^+^ cells (y-axis) between groups 1–4 (x-axis). PBMCs are depicted by dark circles and MMC by open grey circles. Red horizontal bars represent the mean per group examined.

To analyse cellular activation, the expression of CD38 was measured on T cells derived from the peripheral blood and GI tract [12]. Appropriate isotope controls were used to define the gates (data not shown). As similar results were observed for CD4^+^ and CD8^+^ T cell subsets, these data are not described separately here. A striking increase was observed in the expression of CD38 on CD3^+^ T cells of HIV-uninfected IDU subjects (10.5% ± 7.3 CD3^+^CD38^+^ PBMC, and 6.6% ± 4.5 CD3^+^CD38^+^ MMC) compared to healthy controls (3.6% ± 1.8 CD3^+^CD38^+^ PBMC, p<0.001 and 2.2% ± 1.8 CD3^+^CD38^+^ MMC, p<0.001). Among the HIV-1-infected patients, again a notable increase in the expression of CD38 on CD3^+^ T cells was seen among IDU HIV-1 infected subjects (24.0% ± 6.6 CD3^+^CD38^+^ PBMC, and 26.2% ± 17.2 CD3^+^CD38^+^ MMC) as compared to non-injecting HIV-1 infected subjects (7.3% ± 3.8 CD3^+^CD38^+^ PBMC, p<0.001 and 6.2% ± 5.6 CD3^+^CD38^+^ MMC, p<0.001) (Figure 2).

**Figure 3:**
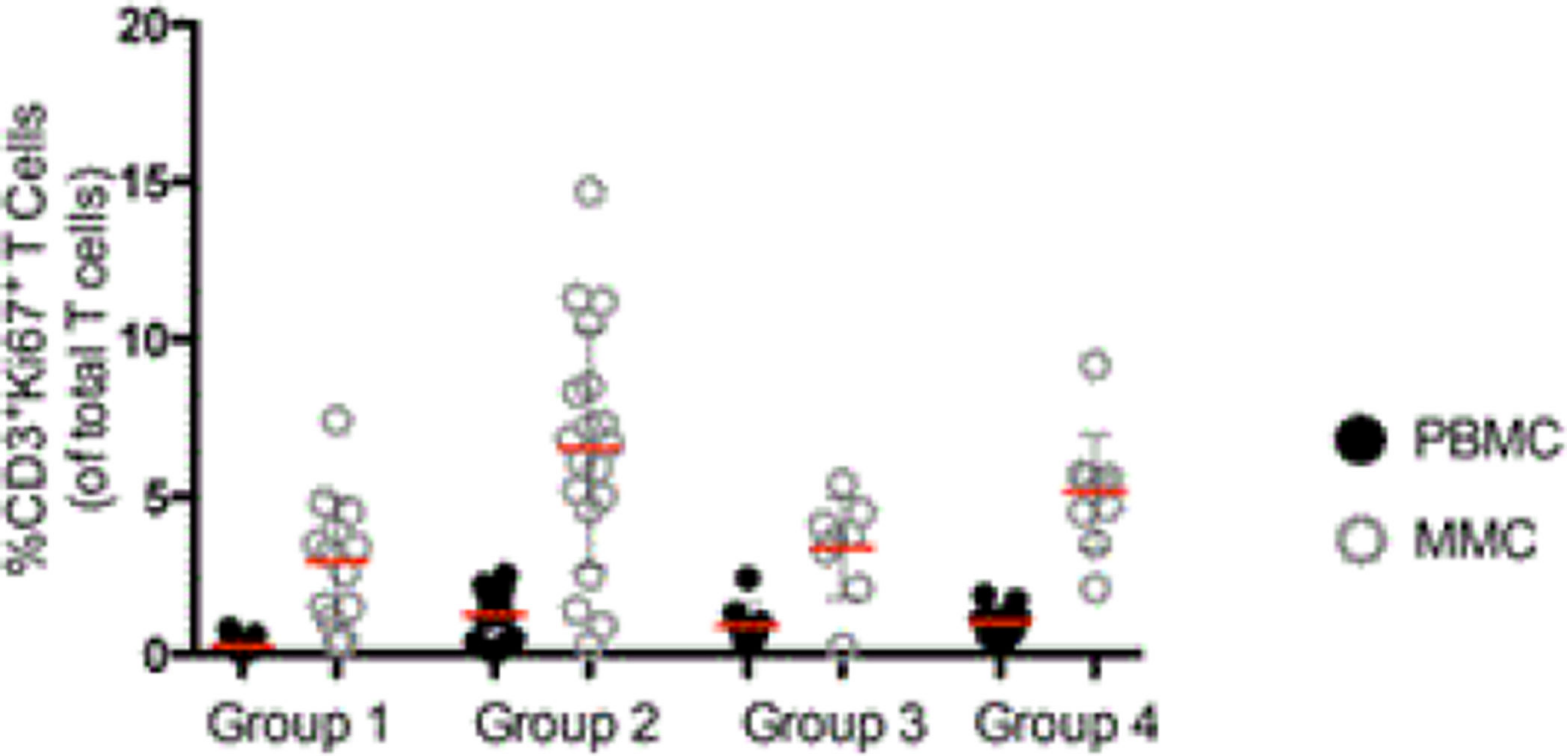
Cumulative data comparing the percentage of CD3^+^Ki67^+^ cells (y-axis) between groups 1–4 (x-axis). PBMCs are depicted by dark circles and MMC by open grey circles. Red horizontal bars represent the mean per group examined

We next went on to examine the expression of Ki67, a marker of cellular proliferation, on CD3^+^ T cells [13]. Again CD4^+^ and CD8^+^ subset data is consistent with that seen in the CD3^+^ population and are not described separately. As was observed with the expression of CD38, a striking increase was observed in the expression of Ki67 on CD3^+^ T cells in group 2, HIV-uninfected IDUs (1.3% ± 0.7 CD3^+^Ki67^+^ PBMC, and 6.5% ± 4.3 CD3^+^Ki67^+^ MMC) compared to group 1 healthy controls (0.3% ± 0.2 CD3^+^Ki67^+^ PBMC, p<0.001 and 2.9% ± 1.9 CD3^+^Ki67^+^ MMC, p<0.001). Among the HIV-infected groups, the differences in the expression of Ki67 were less pronounced between the group 3 non-IDUs (0.9% ± 0.7 CD3^+^Ki67^+^ PBMC, and 3.3% ± 1.6 CD3^+^Ki67^+^ MMC) and the group 4 IDUs (1.0% ± 0.5 CD3^+^Ki67^+^ PBMC, p=0.7 and 5.2% ± 1.8 CD3^+^Ki67^+^ MMC, p<0.05) (Figure 3).

### A soluble marker of immune activation in the peripheral blood of study subjects

**Figure 4:**
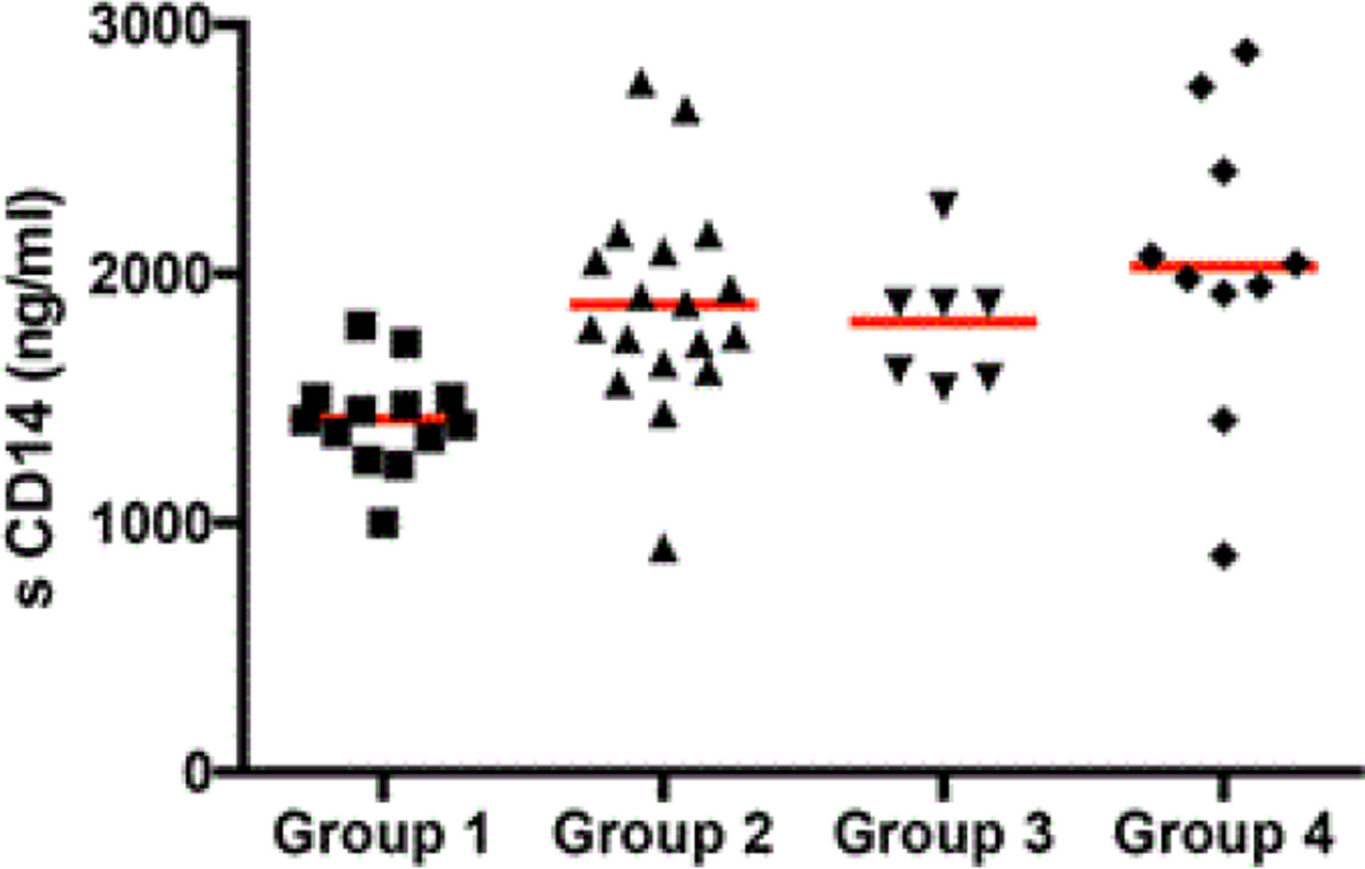
Effect of IDU on plasma levels of sCD14. Levels of sCD14 (ng/ml, y-axis) from Group 1 (non-IDUs, HIV-uninfected, n=13); Group 2 (HIV-1 uninfected IDUs, n=19); Group 3 (HIV-1 infected, viremic, non-IDUs, n=8) and Group 4 (HIV-1 infected, viremic, IDUs n=10) (x-axis). Each of the symbols represents individual data points and the horizontal red line shows the mean for each of the groups.

We measured levels of sCD14, a soluble marker of monocyte activation [13], in the peripheral blood of the four study groups. A significant increase was noted in the level of sCD14 in HIV-uninfected IDUs (Group 2) subjects (1875.6 ng/ml ± 426.6) compared to group 1 healthy controls (1416.0 ng/ml ± 203.5, p<0.001). Among the HIV-infected, differences between group 3, non-IDU (1805.7 ng/ml ± 255.6) and group 4, active IDU (2028.8 ng/ml ± 592.5) subjects were not significant (p=0.3) although the levels of sCD14 were significantly elevated in group 3 (p<0.05) and group 4 (p<0.001) compared to group 1 controls (Figure 4).

### Correlations between injection related behaviours and immune activation

Correlational analyses were conducted to explore the relationship between selected drug use behaviours and immunological parameters. Drug use variables examined were: Injection frequency in prior 30 days, number of years injected, and any sharing of needles or other injection equipment. An interesting pattern that emerged was an association between sharing needles or other injection equipment and immune activation in blood among uninfected IDUs (e.g. r=0.63 between sharing needles and %CD4^+^Ki67^+^ cells (p<0.01) and r=0.50 between sharing paraphernalia and %CD8^+^Ki67^+^ cells, p<0.05).

## Discussion

We conducted this pilot study to examine how IDU affects systemic and mucosal CD4+ T cell depletion and immune activation when superimposed on HIV-1 infection. We found that IDU further increases levels of immune activation as measured by the expression of CD38 in the peripheral blood and the GI tract of HIV-infected injectors compared to HIV-infected non-injectors. Additionally, we found increased levels of proliferating cells within the GI tract of HIV-infected injectors compared to HIV-infected non-injectors. The levels of sCD14 were significantly elevated in the HIV-infected and - uninfected injectors compared to normal volunteers. Although the levels of sCD14 trended higher in the HIV-infected injectors compared to the HIV-uninfected injectors, they did not meet statistical significance. We speculate that in the presence of viremia some effects of active injection may be masked by the effects of untreated HIV-1 infection. We are in the process of characterizing immune parameters both peripherally and mucosally in larger numbers of actively injecting HIV-1 infected subjects, both pre- and post-suppressive antiretroviral therapy, to attempt to better understand the effects of active IDU when superimposed on HIV-1 infection.

Additionally, a number of other important findings have emerged from this pilot project. First, we have demonstrated that it is possible to recruit, retain and study subjects who are active IDUs even with complex invasive procedures like gastrointestinal biopsies. Second, although active IDU in the absence of HIV-1 infection does not result in depletion of CD4+ T lymphocytes per se, it is associated with activation of the immune system. Third, immunological perturbations associated with IDU are not only confined to the peripheral blood but are also seen at mucosal surfaces like the GI tract. We believe this finding of immune activation related to IDU in both blood and tissue is novel and forms the foundation for subsequent investigations already in progress and is discussed below.

Recruitment and retention of IDU in clinical studies is considered challenging [14–18] requiring the use of innovative approaches. We relied on a combination of community outreach by experienced personnel, active and passive recruitment, client education and respect, dedicated follow up, and monetary incentives. Other factors that aided us in retaining IDU subjects were the age of our study cohort (late 30s and early 40s) and a relatively short time between screening and study procedures (approximately 2 weeks). Adopting a combination of the above strategies, we were able to attain a retention rate of over 85% for our study, which included phlebotomy and sigmoidoscopic gastrointestinal biopsies (performed without sedation). Thus, we provide compelling evidence for feasible recruitment and retention strategies to promote meaningful investigation of this understudied patient population.

A seminal report by Wybran and colleagues in 1979 demonstrated that T lymphocytes express opiate receptors [19], providing biological evidence of modulation of the immune system by opioids. Since then, μ, κ and δ- opioid receptors have been identified on human lymphocytes [20–22], platelets [22], macrophages [23], granulocytes [24] and monocytes [25]. Functional effects opioids on the immune system include thymic and splenic atrophy [26,27], inhibition of macrophage phagocytosis [28], inhibition of monocyte and neutrophil chemotaxis to complement derived factors [29] or to chemokines and cytokines [30]. Other studies have shown opioid induced inhibition of antibody production [31], increase in interferon-γ production [32], increase in IL-1 production [33] and TGF-β production [34]. Interestingly, numerous other studies show diametrically opposite results as well [35–37]. In addition to such direct immunological effects, opioids may also exert indirect immune effects through their actions on the central nervous system (each major class of opioid receptors has been cloned from neuronal cells) and the hypothalamic-pituitary-adrenal axis (HPA) [38]. Thus, when looking at an individual as a whole, opioids may affect the brain, the HPA axis or various subpopulations of immune cells in complex ways. This is perhaps one factor to explain the conflicting data on the immunological effects of IDU. Consistent with an overall “immune dysfunction” induced by opioids, animal studies have shown that non virulent strains of *Toxoplasma gondii* can become lethal in morphine sensitized animals [39] and endogenous flora can induce sepsis [40]. Similarly, the virulence of Herpes Simplex Virus [41] and Pastuerella [42] can be potentiated in opioid sensitized animals. The interactions between opioids, the immune system and HIV are harder to investigate. While early epidemiological studies showed reduced survival in HIV-infected IDU patients compared to HIV-infected non IDU controls [43], more recent studies have suggested that progression of HIV-1 infection in IDU as reflected by decline in CD4^+^ T-cell counts, is equivalent to non-IDU controls [44]. Indeed, the data generated in our study demonstrates that IDU does not alter the percentage of CD4^+^ or CD8^+^ T cells, both among HIV-infected or HIV-uninfected individuals.

In addition to numerical changes in T cells, we examined qualitative parameters known to influence HIV-1 disease progression. Guided by our previous studies in acute and early HIV-1 infection, we examined the blood and GI tissue of active IDUs and compared these findings to appropriate controls. The GI tract is the largest immune reservoir in body [45] and is central to the early events in HIV transmission and pathogenesis [1,3]. Furthermore, by allowing translocation of microbial products due to mucosal damage from HIV-1, the GI tract has been found to play an important role in the pathogenesis of chronic HIV-1 infection as well [6]. We chose to focus on cellular and soluble parameters of immunological activation based on conclusive HIV-1 pathogenesis studies. Increased expression of CD38 and HLA-DR on CD4^+^ and CD8^+^ T cells in untreated HIV-1 infection has been associated with rapid disease progression [46,47] and that degree of immune reconstitution following combination antiretroviral therapy is inversely associated with immunological activation [48].

There is a relative paucity of literature describing the link between markers of immune activation, HIV and IDU. In a study by Tran and colleagues, a cohort of 32 HIV-uninfected IDUs had lower levels of naïve CD4^+^ and CD8^+^ T cells and higher levels of CD8^+^CD25^+^ T cells when compared to non-injecting controls. In this study, HIV-1-infected injectors had the highest levels of markers of immune activation. However, no analyses of soluble markers of immune activation were performed and no tissue was obtained from this cohort for analysis [49]. To our knowledge, our study is the first description of mucosal lymphocyte activation associated with IDU. Since activated lymphocytes are preferred targets for HIV infection, we provide a potential biological basis for facilitation of HIV transmission in IDUs in addition to the other known behavioural correlates of transmission.

In seeking to correlate biological observations with behavioural data, we found indications that sharing needles and other injection equipment may be related to immune activation among IDUs who are not HIV-infected, but larger sample sizes are needed to confirm these correlations. It may be that sharing injection-related equipment that is not sterile may expose the IDU to HLA-mismatch or other pathogens and may increase levels of immune activation.

Finally, we must acknowledge the limitations of this study. Firstly, this is a small, proof of concept pilot study as reflected in the large variance in the markers of immunological activation. This somewhat limits the conclusions that can be drawn. Secondly, given the high prevalence of HCV in the IDU population [50] and in this cohort of active IDUs- (74% of HIV uninfected IDUs were HCV^+^ and 90% of HIV-infected patients were HCV^+^), this potentially confounds our results in that we cannot differentiate the effects of non-sterile injection, direct effects of opiates, and that of chronic HCV infection. Although in subgroup analyses of the HIV-uninfected IDUs, no immunological differences were observed among HCV-infected and HCV^−^ uninfected IDUs, the numbers of subjects are small and these findings need to be confirmed in larger controlled studies. Lastly, the findings in this study are correlative and do not establish a direct causality. These findings highlight the need for detailed study of the relationships between injection-related variables and the immune system that are based on larger samples, and can examine the specific drugs used, the route of administration, the impact of injection behaviours, and the role of Hepatitis C infection. This study is underway and will hopefully allow us to differentiate the immunologic effects of non-sterile injection from Hepatitis C infection and direct effect of opioids.

In summary, this pilot study generates provocative and novel findings of enhanced immune activation in the GI tract as well as blood in active IDUs. This in turn suggests that active IDU may have detrimental biological effects and consequences particularly when intersecting with HIV-1 exposure, infection, and perhaps responses to treatment.

## Figures and Tables

**Table 1:** Socio-demographic clinical characteristics of the study volunteers (N=50)

	HIV-uninfected non-IDUs (N=13)	HIV-uninfected IDUs (N=19)	HIV-infected non-IDUs (N=8)	HIV-infected IDUs (N=10)
Gender (% male)	61.5	68.4	87.5	90.0
Age (mean yrs, sd)	39.0 (9.5)	37.4 (8.5)	40.5 (8.6)	42.1 (8.1)
Race/Ethnicity (%)*				
Black	15.4	10.5	75.0	20.0
Hispanic	69.2	73.7	25.0	80.0
White	15.4	15.8	0	0
Education (%)*				
<HS	7.7	42.1	25.0	50.0
GED/HS	23.1	36.8	25.0	50.0
>HS	69.2	21.1	50.0	0
Homelessness (%)	0	10.5	0	0
HIV viral load (copies/ml plasma), mean, (range)	<48	<48	58,409 (13,571–214,696)	63,046 (7,259–111,609)
CD4 count (cells/mm3), mean, (range)	925 (442–1449)	950 (456–2021)	456 (37–645)	264 (9–819)
HCV-infected (number, %)	0/13	14/19 (74)	1/8 (12)	9/10 (90)

* p<0.05;

Note: Significance tests used: Fisher’s exact test for categorical variables and one-way ANOVA for continuous variables.

**Table 2:** Injection & non-injection behaviours by HIV-non-infected vs. HIV-infected IDUs (prior 30 days)

	HIV-non- infected IDUs (N=19)	HIV-infected IDUs (N=10)
Duration of IDU (years, SD)	13.5 (8.7)	19.6 (14.3)
Frequency of injection (#/mo)	160.9 (219.7)	147.1 (127.4)
Drugs injected (%)		
Heroin	89.5	100
Cocaine	63.2	90
Speedball*	47.4	80
Other*	36.8	10
Shared needles/syringes (%)*	57.9	20
Shared other paraphernalia (%)	63.2	70
Non-injecting drug use (%)		
Heroin*	10.5	50
Cocaine/crack	26.3	40
Methadone	73.7	70
Other drugs	63.2	90

* p<0.05,

Note: Significance tests used: Fisher’s exact test for categorical variables and 2-tailed t-test for continuous variables
